# PCSK9 Inhibitors: Focus on Evolocumab and Its Impact on Atherosclerosis Progression

**DOI:** 10.3390/ph17121581

**Published:** 2024-11-25

**Authors:** Maram H. Abduljabbar

**Affiliations:** Department of Pharmacology and Toxicology, College of Pharmacy, Taif University, P.O. Box 11099, Taif 21944, Saudi Arabia; maram.a@tu.edu.sa

**Keywords:** PCSK9 inhibitors, Evolocumab, atherosclerosis

## Abstract

This paper investigates the therapeutic use of PCSK9 inhibitors, particularly Evolocumab, as monoclonal antibodies for the treatment of atherosclerosis based on recent literature reviews. PCSK9 is an outstanding example of a breakthrough in medical science, with advancements in understanding its biological function driving substantial progress in atherosclerosis treatment. Atherosclerotic cardiovascular disease (ASCVD) is a leading global cause of mortality, imposing substantial financial burdens on healthcare systems. Elevated low-density lipoprotein cholesterol (LDL-C), a modifiable risk factor, plays a pivotal role in the development of ASCVD. Emerging treatments such as PCSK9 inhibitors are now being introduced to combat this issue, with the goal of reducing ASCVD risk by directly targeting LDL-C levels. This discovery highlighted the potential of monoclonal antibodies to inhibit PCSK9, thereby enhancing LDL-C receptor activity. This breakthrough led to the development of Alirocumab and Evolocumab inhibitors, which typically reduce LDL-C levels by approximately 50%. This research underscores the importance of PCSK9 inhibitors in treating ASCVD, drawing on evidence from various randomized controlled trials such as FOURIER, ODYSSEY OUTCOMES, and VESALIUS-CV. These trials have also shown that PCSK9 inhibitors are effective and safe for the treatment of several cardiovascular disorders. PCSK9 inhibitors are therefore useful in patients who do not reach their target LDL-C levels when on the highest doses of statins or patients with very high cardiovascular risk who cannot tolerate statins at all.

## 1. Introduction

Cardiovascular disease (CVD) remains the leading cause of death globally, with a significant increase in age-related chronic conditions. In 2021, CVD caused approximately 20.5 million deaths, a 60% increase from 1990 [1]. Atherosclerosis, the buildup of plaques in blood vessels, is a key contributor, leading to heart attacks, strokes, and peripheral vascular disease [2]. It is closely linked to low-density lipoprotein (LDL), which transports cholesterol in the blood and is influenced by hypertension, smoking, diabetes, inflammation, and clonal hematopoiesis [3].

CVD-related deaths have risen since 1990, with ischemic heart disease increasing by 72%, cardiomyopathy and myocarditis by 48%, and hypertensive heart disease by 92%. Stroke cases have also grown by 47%, indicating a shift in cardiovascular health [4]. In 2015, the FDA approved two PCSK9 inhibitors, Alirocumab and Evolocumab, for adults with familial hypercholesterolemia (HeFH) or atherosclerotic cardiovascular disease (ASCVD) who need further LDL-C reduction [5,6]. These medications, used with diet changes and statins, provide an advanced approach for significantly lowering LDL-C. However, statins alone often fail to meet LDL-C targets, highlighting the need for combined therapies like Ezetimibe with PCSK9 inhibitors [7].

Atherosclerosis is traditionally linked to high cholesterol [8], although factors like hypertension, smoking, diabetes, and high triglycerides also contribute [9,10]. Excess LDL-C accumulates in arterial walls, leading to plaque formation [11,12]. Oxidized LDL triggers an inflammatory response, causing smooth muscle cells to migrate and release cytokines that promote further inflammation [13,14,15].

LDL-C control variability affects cardiovascular outcomes, with differences in adherence and individual response. Evolocumab, a PCSK9 inhibitor, can reduce LDL-C by about 60%, either alone or combined with statins and Ezetimibe. These therapies significantly reduce cardiovascular events, but real-world data on maintaining LDL-C reductions remain limited [16]. Atherosclerosis can be asymptomatic but often leads to coronary artery disease, heart failure, aneurysms, and stroke, with symptoms like chest pain, irregular heartbeats, fatigue, and light-headedness [17,18,19].

Preventing atherosclerosis involves lifestyle changes like a balanced diet, physical activity, smoking cessation, and stress management [20]. Recommendations include limiting red and processed meats, eating fish and eggs, and consuming ≥400 g of vegetables and fruits daily to support cardiovascular health [21,22,23,24,25].

The 2019 American College of Cardiology (ACC) and American Heart Association (AHA) guidelines for ASCVD prevention are multidisciplinary. Adults should engage in 150 min of moderate exercise weekly. Those with type 2 diabetes should adopt a heart-healthy diet to improve glucose control. Moderate-intensity statins are advised for adults with high cholesterol and a moderate CVD risk (7.5% to < 20%), aiming for a 30% LDL-C reduction. High-risk individuals should aim for a 50% reduction, with the highest statin doses for patients with LDL-C levels ≥190 mg/dL or aged 20–75. Additional measures include low sodium intake, increased physical activity, and smoking cessation support [4,26,27,28,29].

This study investigates PCSK9 inhibitors, particularly Evolocumab, in treating atherosclerosis by managing LDL-C levels with statin therapy. It reviews recent trials, including the FOURIER trial, which assessed Evolocumab’s effectiveness in reducing ASCVD events in high-risk patients. The paper highlights the need for supportive policies and affordable access to these essential medications.

## 2. The Evolution and Significance of PCSK9

Atherosclerosis is a major cause of heart-related deaths worldwide, and researchers are focusing on PCSK9 due to its ability to lower cholesterol [30,31,32,33,34], even in individuals already taking statins. PCSK9 is important for diagnosing and treating inherited hypercholesterolemia and other cholesterol-related diseases [35]. Tang et al. [36] reviewed 2474 studies on PCSK9 and atherosclerosis from 2006 to 2023, emphasizing its role in reducing cholesterol in statin users. Most studies came from the U.S., with atherosclerosis publishing the most. Earlier research mainly focused on PCSK9′s effect on cholesterol, while recent advancements include gene editing techniques like CRISPR and new treatments for heart diseases, advancing future research in heart health and PCSK9.

Epidemiological studies support the critical role of LDL-C in atherosclerosis formation. Reducing LDL-C, shown in several trials, decreases the risks of myocardial infarction, stroke, and mortality. Discovered in 2003, PCSK9 targets receptors like LDLR, increasing circulating LDL-C levels in the bloodstream. High LDL-C is often hereditary, with genetic variations causing elevated LDL-C, especially in familial hypercholesterolemia (FH) [37,38,39,40].

Research into the PCSK9 pathway has led to anti-PCSK9 treatments, such as monoclonal antibodies, which effectively lower LDL cholesterol but are costly and require biweekly injections. Another FDA-recognized method is RNA interference, reducing PCSK9 levels with semiannual injections [41,42,43]. The discovery of PCSK9′s crucial role has led to the rapid development of inhibitors, particularly monoclonal antibodies, which lower LDL-C by 50–70%. These medications are mainly used for patients with FH, statin intolerance, or hyperlipidemia. Inclisiran, an RNA-based therapy, degrades PCSK9 mRNA, offering similar effectiveness to monoclonal antibodies but with longer action. However, the extended safety, cost-effectiveness, and widespread use of PCSK9 inhibitors in preventing coronary events need further validation [42,44].

PCSK9 inhibitors and siRNA molecules, which suppress PCSK9 expression in the liver, are now widely used to manage elevated cholesterol levels. These therapies, often used with statins, reduce atherosclerosis incidence by at least 20%. Since 2008, PCSK9 has also been linked to inflammation and cancer metastasis, highlighting its broader role in health beyond regulating LDL-C [45,46].

## 3. PCSK9 Inhibitors

PCSK9, belonging to the subtilisin protease category, an enzyme involved in protein digestion, was first identified in the brain by a Canadian research institute in 2003 and subsequently designated as PCSK9 according to established naming conventions [47]. The PCSK9 protein forms through the linkage of a signal protein fragment, propeptide, reaction site, and C-terminal [48] and is situated in the chromosome 1p32 region, predominantly found in the hepatorenal and intestinal parts [49]. Research in controlled lab settings and observations from genetic mutations indicate that this enzyme family plays specific physiological roles by modifying and controlling various proteins, influencing their activation or deactivation. Elevated LDL-C levels in the blood significantly contribute to atherosclerosis, underscoring the importance of lowering LDL-C, wherein PCSK9 plays a crucial role in this molecular mechanism [50].

PCSK9 inhibitors are primarily used in clinical settings for treating high cholesterol, atherosclerosis, and related heart-related conditions, showing substantial efficacy and potential as alternatives to statins and Evolocumab [51,52]. These inhibitors can mitigate endothelial dysfunction, prevent plaque buildup, and maintain heart muscle integrity [53,54]. PCSK9 is implicated in cardiovascular disease progression through multiple mechanisms, including the degradation of LDL receptors, which elevates circulating LDL levels [55,56]; interaction with Toll-like receptors, leading to inflammatory responses [57]; and binding with the CD36 receptor, promoting blood clotting and platelet activation [58].

## 4. The Molecular Pathways of PCSK9

PCSK9 influences heart disease progression through multiple pathways, such as LDL receptor downregulation, which increases LDL levels, inflammation through engagement with Toll-like receptors (TLRs), and clot formation by interacting with platelet glycoprotein IV (CD36) to activate platelets. PCSK9 primarily degrades the LDL receptor, a key process in lowering LDL-C levels in the bloodstream. LDL receptors help internalize LDL-C on liver cell membranes, but when PCSK9 binds to them, the complex is degraded in lysosomes, preventing recycling of the receptors. This leads to higher circulating LDL-C levels, which contribute to atherosclerosis and cardiovascular diseases like ischemic heart disease [43,55,56,57,58,59,60,61,62,63,64,65].

PCSK9 binds LDL receptors in two phases: a rapid attachment with a half-life of 5–10 min and a slower binding phase with a half-life of about 1.5 h. Recent studies in laboratory and living organism models have explored how PCSK9 directs LDL receptors for internalization and degradation after binding [66,67]. Additionally, PCSK9 interacts with TLR4, activating the NF-KB pathway and promoting inflammation through increased secretion of inflammatory factors. This mechanism contributes to endothelial dysfunction and worsens conditions like sepsis, highlighting PCSK9′s role in inflammatory disease progression [68].

PCSK9 also binds to CD36, a platelet glycoprotein involved in the uptake of LDL and other substances. This binding enhances platelet activation and reduces lipid uptake, promoting inflammation and contributing to atherosclerosis progression. Studies show that PCSK9 inhibitors decrease LDL-C, oxidized LDL (ox-LDL), and thrombosis markers in patients with inherited high cholesterol. PCSK9′s role in platelet activation involves promoting reactive oxygen species (ROS) levels and thrombosis, which can lead to arterial clotting disorders like myocardial infarction and ischemic stroke [53,69].

Monoclonal antibodies against PCSK9, such as Alirocumab and Evolocumab, are designed to target and inhibit PCSK9, reducing LDL cholesterol levels significantly. These therapies are particularly useful for patients who do not respond to statins, experience side effects, or have genetic predispositions to high cholesterol. Although statins are the primary treatment for many individuals, they do not always achieve sufficient cholesterol reduction. PCSK9 inhibitors offer an effective alternative, showing promising results in reducing cardiovascular events. These monoclonal antibodies are administered via subcutaneous injections at intervals of up to one month, with ongoing studies focusing on their long-term benefits and safety. This new class of medications, along with other monoclonal antibody treatments, is advancing the prevention and management of cardiovascular disease [70].

## 5. Monoclonal Antibodies Against PCSK9

PCSK9 inhibitors are monoclonal antibodies generated via cell culture methods to specifically target and bind PCSK9. These inhibitors, such as Alirocumab and Evolocumab, effectively reduce LDL cholesterol and are particularly useful for patients who do not respond to statins, experience side effects or have genetic predispositions to high cholesterol. While statins are the primary treatment for high cholesterol, they may not be sufficient for high-risk individuals. Ongoing studies aim to confirm the long-term safety and benefits of PCSK9 inhibitors in reducing cardiovascular events. These antibodies are injected beneath the skin monthly and represent a promising new treatment option for preventing cardiovascular disease [70].

## 6. Evolocumab: A Key Therapy for Reducing Atherosclerosis Risk

**Figure 1:**
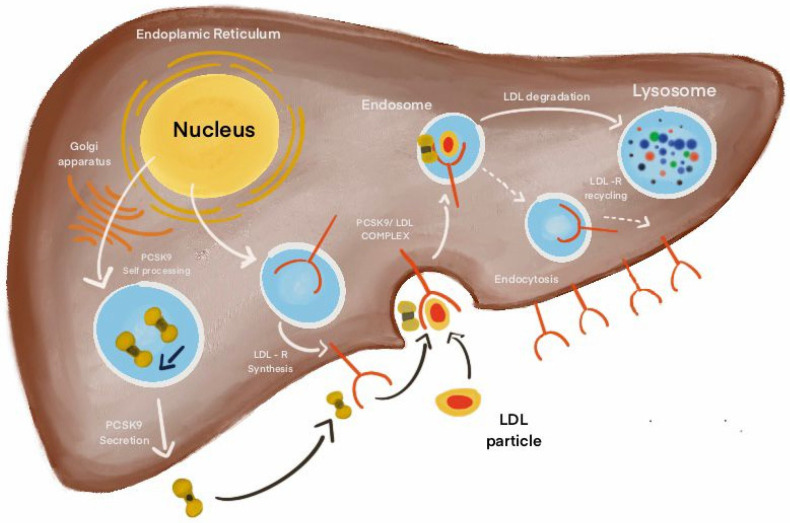
The role of PCSK9 (Evolocumab) inhibitors in CVD.

PCSK9 inhibition is facilitated by drugs such as Evolocumab, and it may be achieved through the following processes. Evolocumab is a fully human monoclonal antibody that targets PCSK9. Its primary function is to prevent PCSK9 from binding to LDL receptors, which enhances the uptake of LDL-C. In the liver, LDL receptors on liver cells facilitate the uptake and removal of LDL-C from the bloodstream. The enzyme PCSK9 controls the expression of these LDL receptors by binding to them on the cell surface. When PCSK9 binds to the LDL receptor, it triggers the receptor’s internalization and subsequent breakdown. Preventing this interaction leads to higher levels of LDL receptors on the cell surface. This increase enhances the liver’s ability to capture LDL-C from the blood, ultimately reducing LDL-C levels in the bloodstream [71]. As LDL-C levels drop, the accumulation of cholesterol in arterial walls decreases, resulting in reduced formation of atherosclerotic plaque. Consequently, with less plaque constricting blood flow in the arteries, cardiovascular health is improved, lowering the risk of severe events such as heart attacks and strokes (see Figure 1). 

### 6.1. Findings from Clinical Trials Using PCSK9 Inhibitors

PCSK9 inhibitors are a new class of cholesterol-lowering drugs that offer an additional treatment option for patients struggling to achieve low LDL-C goals. These inhibitors, including monoclonal antibodies like Evolocumab and Alirocumab, and Inclisiran, which inhibits PCSK9 synthesis, effectively lower LDL-C and other harmful lipid–protein complexes (see Table 1). Recommended for patients with inherited high cholesterol or high risk of atherosclerosis, PCSK9 inhibitors help those with greater risk achieve their LDL-C targets. Recent research confirms the long-term efficacy and safety of Evolocumab, while ongoing trials explore new therapeutic applications and evaluate cost-effectiveness in reducing residual risk in atherosclerosis prevention.

Choi et al. [82] conducted a meta-analysis to assess the risks associated with the use of Alirocumab and Evolocumab, two PCSK9 inhibitors known for lowering cardiovascular risk. The study compared these drugs with control groups receiving either a placebo or Ezetimibe (a cholesterol-lowering medication) to evaluate their safety and effectiveness. The analysis did not reveal significant differences in adverse event risks between the groups. Research indicates that Alirocumab lowers LDL-C levels by 39 to 62%, while Evolocumab reduces LDL-C by 47 to 56% [73,83].

A recent meta-analysis investigating the effects of PCSK9 inhibitors like Evolocumab on lipid profiles and safety in patients with genetically high cholesterol levels reviewed 10 studies with 1489 patients. The findings showed significant reductions in LDL-C levels. Specifically, PCSK9 inhibitors such as Evolocumab and Alirocumab lowered LDL-C by an average of 49.59% compared to baseline levels. These results align with prior randomized trials, which show that these inhibitors can achieve LDL-C reductions ranging from 50 to 60%. Despite some researchers advocating for moving beyond LDL-C as the primary marker for assessing atherosclerosis risk, LDL-C remains a well-established clinical target. Numerous studies have shown that reducing LDL-C levels is linked to a decrease in cardiovascular risk [84,85]

In another study based on the HEYMANS registry, researchers explored the use of Evolocumab in practical clinical settings across twelve European countries. The goal was to understand patient adherence to Evolocumab treatment and examine the changes in LDL-C levels over 30 months. Results revealed that Evolocumab significantly reduced LDL-C levels by an average of 58% within three months, with this effect sustained throughout the study. A significant majority of patients continued Evolocumab treatment at both twelve and thirty months, and nearly 85% of patients whose LDL-C levels were measured showed at least a 30% reduction, with about 60% experiencing a reduction of 50% or more compared to their baseline levels [75].

PCSK9 inhibitors, especially Evolocumab, show promise in improving the safety and effectiveness of LDL-C-lowering therapies for patients at high cardiovascular risk. The FOURIER trial and its extension, FOURIER-EOLT, demonstrated significant reductions in cardiovascular risk with Evolocumab in patients with atherosclerosis, particularly those with multivessel coronary artery disease (MVCAD). Over five years, MVCAD patients saw up to a 38% reduction in cardiovascular risk, while patients without MVCAD experienced up to a 28% reduction in risk [74]. PCSK9′s role in cholesterol metabolism is also linked to inflammatory responses that can worsen tissue damage, particularly after coronary injury [86,87].

Ziogos et al. [75] conducted a study on the early development of cardiac inflammation following acute myocardial infarction (MI) and the effect of PCSK9 inhibition using Evolocumab. Using fluorodeoxyglucose positron emission tomography (FDG-PET) scans, they measured inflammation in patients immediately after MI and again after 30 days. Their randomized, placebo-controlled trial showed that Evolocumab significantly reduced cardiac inflammation compared to a placebo when administered shortly after MI [75].

Rosenson et al. [76] performed a double-blind, placebo-controlled trial involving 41 subjects with atherosclerotic cardiovascular disease (ASCVD) and comorbidities. These participants, already on high-dose statins, were randomly assigned to receive either Evolocumab or a placebo. Over 12 weeks, Evolocumab reduced LDL-C by 68.8% at two weeks and 52.8% at twelve weeks. The study also revealed distinct gene expression differences between the Evolocumab and placebo groups, highlighting changes in immune cell activity and stress responses [76].

Evolocumab, a monoclonal antibody that inhibits PCSK9, not only lowers LDL-C levels but also reduces the occurrence of major adverse cardiovascular events in individuals with established ASCVD, which includes prior MI, stroke, and peripheral vascular disease [88,89,90]. The drug has shown no significant safety concerns in these groups. However, its impact on cardiovascular outcomes in patients with lower cardiovascular risk, who have no history of heart attack or stroke, has not been thoroughly studied.

The VESALIUS-CV trial is a large, international, double-blind study evaluating Evolocumab’s effectiveness in 12,301 patients with coronary artery disease (CAD) or diabetes and cardiovascular complications but no history of MI or stroke. These patients exhibit elevated cholesterol markers, including LDL-C of 90 mg/dL or higher, non-HDL-C of 120 mg/dL or higher, and apolipoprotein B of 80 mg/dL or higher [77]. The study aims to determine if biweekly doses of Evolocumab can reduce the risk of CAD death, heart attacks, strokes, and the need for arterial revascularization. The patients will be followed for at least 4.5 years to assess Evolocumab’s long-term effects on major heart events [77].

In the FOURIER trial, patients with chronic CAD already on statin therapy were randomized to receive either Evolocumab or a placebo [74]. The study followed these patients for an average of 2.2 years. The data showed that Evolocumab significantly reduced LDL-C levels and decreased the risk of primary cardiovascular events by 17% and secondary events by 24% in patients with metabolic syndrome (MetS). This trial supports the combination of Evolocumab with statin therapy as an effective approach to reducing cardiovascular risk in patients with both MetS and ASCVD [74].

A separate analysis of the FOURIER trial showed that Evolocumab reduced LDL-C levels by 59% and lowered the occurrence of major vascular events. This study focused on cerebrovascular events, particularly in patients who had a prior stroke. The research, involving over 27,000 patients with established atherosclerosis and high LDL cholesterol despite statin treatment, revealed a significant reduction in stroke incidence in those receiving Evolocumab compared to the placebo group. Similarly, a meta-analysis by Cordero et al. [80] involving 81,700 patients found that PCSK9 inhibitors, including Evolocumab, reduced heart attacks by 19% and strokes by 25% compared to control groups.

Research from the FOURIER trial also revealed that Evolocumab lowered LDL-C levels more significantly in Asian patients (66% vs. 58% in non-Asian patients). Both groups experienced similar reductions in cardiovascular event risks, and no increase in serious adverse events was observed among Asian patients. Evolocumab’s efficacy in reducing primary cardiovascular endpoints was consistent across both Asian and non-Asian groups, with no significant differences in outcomes (*p* interaction = 0.55) [77].

The impact of PCSK9 inhibitors on endothelial function in heart attack survivors is not yet fully understood. Rexhaj et al. [91] studied the effects of adding Alirocumab to high-dose statin therapy on flow-mediated dilation (FMD) and its relationship with coronary artery disease in the PACMAN-AMI trial. This randomized, double-blind study compared Alirocumab with a placebo in combination with rosuvastatin. The results showed that Alirocumab significantly reduced LDL-C levels compared to the placebo (*p* < 0.001) [92].

PCSK9 inhibitors also appear to have potential anti-inflammatory effects, though the evidence is still unclear. Luo et al. [93] conducted a systematic review and meta-analysis to evaluate the anti-inflammatory effects of PCSK9 inhibitors. The review included studies that examined changes in inflammatory markers such as C-reactive protein, IL-1β, IL-6, and TNF-α in adult patients with atherosclerosis. While statins are known for lowering cholesterol and reducing inflammation, PCSK9 inhibitors may have additional benefits by stabilizing vulnerable plaques and potentially reducing vascular inflammation, suggesting that their effects go beyond lipid-lowering alone [93].

### 6.2. Clinical Studies Evaluating Patient Health Outcomes

Research indicates that hypolipidemic therapies, particularly PCSK9 inhibitors, are designed for long-term use to lower the risk of atherosclerotic cardiovascular disease (ASCVD). However, evaluating their long-term efficacy and safety is challenging due to the typically short duration of clinical trials, which may not fully capture the benefits and risks of extended use. The ODYSSEY OUTCOMES trial focused on patients recently experiencing acute coronary syndrome, while the FOURIER trial included those with established cardiovascular disease.

The ODYSSEY OUTCOMES trial, led by Goodman et al. [94], examined the impact of Alirocumab in 18,924 patients over five years. Results showed that Alirocumab significantly reduced LDL-C levels and lowered critical cardiovascular outcomes (CEOs) without increasing side effects, except for mild injection-site reactions. Subgroup analysis indicated that patients who received Alirocumab had sustained cholesterol reduction and a lower risk of cardiovascular death compared to the placebo group. The safety assessment revealed a slightly lower percentage of adverse events in the Alirocumab group (78.3%) compared to the placebo (80.2%), with serious side effects occurring in 27.5% of patients receiving Alirocumab versus 29.4% in the placebo group. Adverse effects like new-onset diabetes and liver enzyme elevation were similar in both groups.

PCSK9 inhibitors also impact Lipoprotein(a) [Lp(a)], a lipoprotein linked to heart disease. These inhibitors reduce Lp(a) levels by 20–30%, but their exact mechanism remains unclear. While PCSK9 inhibitors lower LDL-C levels more significantly than Lp(a), the two are not always reduced in a consistent ratio. In Phase 3 trials of Evolocumab, it was found that 18.4% of patients had a significant reduction in LDL-C without a corresponding decrease in Lp(a), particularly in those with higher baseline Lp(a) levels. This suggests that PCSK9 inhibitors may reduce Lp(a) through mechanisms independent of LDL-C lowering, especially in individuals with elevated Lp(a) levels [95,96].

A study by Mahmood et al. [97] investigated discordant responses to Alirocumab, particularly among patients with familial hypercholesterolemia (FH). The research analyzed data from 10 randomized trials within the ODYSSEY Phase 3 clinical program, focusing on high-risk cardiovascular patients. The study found a high occurrence of discordant responses in LDL-C and Lp(a) levels following treatment with Alirocumab. These results imply that Alirocumab reduces plasma Lp(a) through mechanisms distinct from LDL receptor-mediated clearance, highlighting the complexity of managing lipid profiles with PCSK9 inhibitors [97].

## 7. Long-Term Efficacy and Safety of Evolocumab: Insights from Clinical Studies

In their meta-analysis of 39 randomized controlled studies, Guedeney et al. [98] assessed the efficacy of Alirocumab and Evolocumab in patients with abnormal lipid levels or ASCVD, involving 66,478 patients, with 35,896 receiving PCSK9 inhibitors. Over an average follow-up of 2.3 years, PCSK9 inhibitors did not significantly impact cardiovascular mortality but were associated with reduced risks of MI, ischemic stroke, and coronary artery bypass graft compared to control groups. Importantly, the use of PCSK9 inhibitors did not increase the risk of neuropsychological issues, hepatic enzyme elevations, muscle breakdown syndrome, or recently diagnosed diabetes mellitus.

For individuals with familial hypercholesterolemia (FH) who face elevated ASCVD risks due to high cholesterol levels, anti-PCSK9 monoclonal antibodies have been in use since 2015 in Belgium. A study involving 239 FH patients, 85% of whom were extremely vulnerable to cardiovascular disease, showed a 54% decrease in LDL cholesterol within the first year of treatment, maintained over an average of 3.0 years. Notably, half of the participants achieved their cholesterol targets, with 93% also taking statins alongside the PCSK9 inhibitors. The study concluded that PCSK9 monoclonal antibodies are safe and effective for reducing LDL cholesterol levels in FH patients, especially when used in combination with statins [98] (see Table 2).

Koren et al. [99] conducted the OSLER 1 trial to investigate the long-term effects of Evolocumab over nearly five years. The trial showed that patients receiving Evolocumab experienced a 56% reduction in LDL-C. The severity of side effects was similar to those in the standard treatment group, with minimal discontinuations due to adverse effects. No harmful antibodies were detected in patients treated with Evolocumab, demonstrating that it is safe and well tolerated for long-term cholesterol management.

## 8. PCSK9 Inhibitor Access Challenges: Understanding the Landscape and Its Cost-Effectiveness

The development of PCSK9 monoclonal antibodies has highlighted challenges faced by U.S. doctors and patients in obtaining these treatments. Recent studies suggest practical solutions to simplify access for eligible patients, underscoring the broader issue of access to new medications. This issue not only affects PCSK9 inhibitors but could also influence future treatments, emphasizing the need to resolve these barriers to foster innovation and ensure patients receive the latest treatments, which are vital for maintaining effective physician–patient relationships [102].

Pharmaceutical economics plays a key role in these challenges, particularly the high cost of PCSK9 inhibitors, which is a significant barrier to approval. An overestimation of the number of patients needing these drugs and incorrect economic forecasts, such as the Institute for Clinical and Economic Review (ICER) predicting costs of USD 1.2 billion in the first year, contributed to heightened caution among healthcare payers and complex approval processes. In reality, the expenditure was only USD 83 million, much lower than forecasted, which could explain recurrent denials and the subsequent need for lengthy appeals [103].

The high cost of PCSK9 inhibitors, ranging in the thousands annually for medications like Alirocumab and Evolocumab, further complicates their approval. Justification for their use is generally based on two conditions: patients with established cardiovascular disease who do not reach target LDL-C levels on maximum statin therapy or those who are statin-intolerant [104].

Atherosclerosis, driven in part by hyperlipidemia, imposes significant health and economic burdens worldwide. Statins are the primary treatment to reduce LDL-C and CVD risks; however, some patients experience resistance, limiting their effectiveness. PCSK9 inhibitors offer a promising alternative, with strong cholesterol-lowering abilities, though they are administered biweekly or monthly due to their injectable nature [105].

A U.S. study assessing patient experiences with PCSK9 inhibitors found that after insurance approval, only a small percentage (2.3%) could not afford the medication. The majority of patients found the costs manageable, paying an average of USD 57 monthly out-of-pocket. This contrasts with prior studies showing that almost one-third of patients never filled their prescriptions due to cost [106,107].

The economic value of treatments like PCSK9 inhibitors is typically evaluated through quality-adjusted life years (QALYs) and incremental cost-effectiveness ratios (ICERs). Compared to statins, PCSK9 inhibitors are more expensive, with costs ranging between USD 141,700 and USD 450,000 for each additional year of good-quality life gained [108]. However, the overall cost-effectiveness of PCSK9 inhibitors, particularly Evolocumab and Alirocumab, is generally more favorable than traditional lipid-lowering agents. For instance, in Germany, Evolocumab costs EUR 62,722 with a QALY of 0.55, while Alirocumab costs EUR 87,002 with a QALY of 0.87. In the U.K., Evolocumab costs GBP 45,279 (QALY: 0.53), and Alirocumab costs GBP 46,375 (QALY: 0.86) [109] (see Table 3).

## 9. Comparison of Evolocumab vs. siRNA

High cholesterol, particularly abnormal LDL-C levels, increases the risk of cardiovascular disease (CVD), leading to heart attacks and strokes. Reducing LDL-C has been shown to lower CVD risk. Prior to Inclisiran, cholesterol-lowering medications such as statins, niacin, bile acid sequestrants, fibrates, and monoclonal antibodies like Alirocumab and Evolocumab were used. Statins and similar drugs target enzymes involved in cholesterol assembly and reduce arterial stiffening, decreasing heart attack and stroke risk.

Ebenezer et al. [109] overview siRNA therapies, focusing on Inclisiran and its role in treating lipid disorders. Synthetic small interfering RNAs (siRNAs) can silence specific genes to stop the production of certain proteins. One key target is PCSK9, which reduces the LDL-C receptor, controlling cholesterol levels. Both monoclonal antibodies and siRNAs aim to manage lipid disorders and improve CVD outcomes by targeting PCSK9. While monoclonal antibodies bind to receptors or proteins in the blood, siRNAs face challenges in entering cells. Researchers use N-acetylgalactosamine (GalNAc) conjugates to facilitate siRNA delivery specifically to liver cells, where cholesterol regulation occurs. Inclisiran is a GalNAc-conjugated siRNA that blocks PCSK9 production, with the significant benefit of needing administration every 3 to 6 months, less frequently than monoclonal antibodies, which require more frequent dosing [109] (refer to Table 4 for additional comparisons).

## 10. Limitations of Using PCSK9 Inhibitors

Dyslipidemia is a multifactorial disorder characterized by increased levels of lipids, cholesterols, and triglycerides in the bloodstream, which are associated with an increased risk of heart disease. Treatments have evolved over the years; previously, only statins were available, while now people turn to other medications such as PCSK9 inhibitors like Evolocumab, Alirocumab, and Inclisiran for lipid level improvement. Although current guidelines recommend that PCSK9 inhibitors should be taken by high-risk patients or patients who cannot take statins, their prescription is hampered due to high costs. These studies demonstrate that the abovementioned inhibitors greatly reduce LDL cholesterol numbers; however, long-term side effects and possible interference with other CVD risk factors are not yet fully understood. Cost-effectiveness studies have shown mixed results; some countries have concluded that it is cheaper for some patients, while others are of a different opinion. In general, PCSK9 inhibitors are effective at lowering LDL cholesterol and may provide benefits in the highest-risk patients, but the cost remains a concern [104].

PCSK9 inhibitors, which were introduced to the market in 2015, are significantly more potent than statins in reducing LDL-C levels. As noted, they act by binding to the PCSK9 enzyme that otherwise promotes the degradation of LDL receptors in the liver cells in order to increase the uptake of LDL. There are new classes of drugs called monoclonal antibodies, such as Alirocumab and Evolocumab, which, when administered, can lower the LDL-C by up to 60% in dyslipidemia patients. These medications are effective at reducing cholesterol levels and mitigating CVD risk factors, although they can cause side effects. The agents that are commonly used may induce severe muscular problems in patients through statins and fibrates, and niacin in particular has many side effects, ranging from muscle pain to liver issues. While PCSK9 inhibitors are less likely to cause severe side effects, they need to be administered more frequently. Therefore, there is an urgent need to develop new therapies that bring about a reduction in LDL-C but are devoid of significant risks [110,111].

## 11. Conclusions

Monoclonal antibodies are pivotal among PCSK9 inhibitors, having proven effective at reducing LDL-C levels and mitigating severe adverse cardiovascular events in numerous studies and trials. They offer enduring benefits, proving particularly advantageous for patients who are intolerant of statins or those with familial hypercholesterolemia. These new monoclonal antibody inhibitors are designed to complement statins, helping high-risk cardiovascular patients achieve target LDL-C levels even when on maximum statin doses or unable to tolerate any statin dose. They are considered very safe and have shown no major associated issues thus far. When used wisely, these novel inhibitors are expected to enhance life expectancy and quality of life significantly. Yet, ongoing research is needed to better understand their long-term effects, manage high costs, and refine patient selection criteria.

## Figures and Tables

**Table 1:** Summary of recent advances in clinical trials utilizing PCSK9 inhibitors.

Study Type	Agents	Results	Ref.
Meta-analysis	Alirocumab and Evolocumab	The treatment decreased LDL-C levels by an average of 49.59% (with a confidence interval of −55.5% to −43.67%) compared to the placebo.	Ghasempour et al. [72]
Real-world European Evolocumab study: insights from HEYMANS	Evolocumab	LDL-C levels decreased by 58% within 3 months of starting Evolocumab and remained consistently lower over the subsequent 30 months.	Ray et al. [73]
FOURIER extended open-label trial (FOURIER-EOLT) study	Evolocumab	Evolocumab treatment led to greater cardiovascular risk reductions in patients with multivessel disease (MVD), with risk reductions increasing over time to 37–38% in MVD patients and 23–28% in non-MVD patients.	McClintick et al. [74]
Placebo-controlled, randomized trial	Evolocumab	Achieved significant results in the reduction in cardiac inflammation compared to placebo.	Ziogos et al. [75]
Double-blind, placebo-controlled trial	Evolocumab	Evolocumab achieved a reduction in LDL-C levels by 68.8% (*p* < 0.0001) at 2 weeks and by 52.8% (*p* < 0.0001) at 12 weeks.	Rosenson et al. [76]
VESALIUS-CV trial	Evolocumab	Latest ongoing (results pending).	Bohula et al. [77]
FOURIER randomized clinical trial	Evolocumab	Evolocumab achieved reductions in LDL cholesterol levels across both groups.	Deedwania et al. [78]
FOURIER randomized, double-blind trial	Evolocumab	Evolocumab achieved a notable decrease in total strokes (1.5% versus 1.9%) and ischemic strokes (1.2% versus 1.6%) compared to the placebo.	Giugliano et al. [79]
Recent meta-analysis of randomized clinical trials	Evolocumab, Alirocumab, and bococizumab	Treatment with PCSK9 inhibitors achieved a 19% lower risk of heart attacks and a 25% lower risk of strokes compared to controls.	Cordero et al. [80]
FOURIER trial	Evolocumab	The results showed that Evolocumab effectively reduces LDL-C levels and demonstrates comparable efficacy in reducing cardiovascular events among Asian individuals.	Keech et al. [81]

**Table 2:** Long-term efficacy and safety of Evolocumab.

References	Study Design	Participants	Duration	Efficacy (LDL-C Reduction)	Key Findings (Side Effects/Safety)
[94]	Meta-analysis of 39 randomized controlled trials (RCTs)	66,478 in total, 35,896 receiving PCSK9 inhibitors	2.3 years (avg follow-up)	- Reduced risk of MI, ischemic stroke, and coronary artery bypass graft	No significant impact on cardiovascular mortality; no increased risk of neuropsychological issues, liver enzyme elevations, muscle breakdown syndrome, or new-onset diabetes.
[98]	Real-world study on FH patients	239 patients with FH	3 years	54% reduction in LDL-C in the first year, maintained over the study period	PCSK9 inhibitors are safe and well tolerated, with no significant adverse effects; 93% of patients used PCSK9 inhibitors with statins; half met EAS cholesterol targets.
[99]	49 RCTs (updated safety evaluation)	66,068 participants	N/A	N/A	No difference for adverse events (AEs); Alirocumab reduced diabetes-related AEs; no difference in neurocognitive and neurological AEs; Evolocumab did not show benefits in diabetes.
[100]	Randomized controlled trial	Evolocumab group vs. standard medical treatment	5 years (1-year RCT, 4-year extension)	56% reduction in LDL-C over 5 years	Similar side effect profile to standard treatment group; no harmful antibodies detected; few patients discontinued due to side effects.
[101]	9 studies (RCTs)	1836 participants	N/A	Significant LDL-C and total cholesterol reductions	Significant reductions in LDL-C and total cholesterol, as well as regression of coronary atheroma volume in PCSK9 inhibitor groups, particularly in patients on statins.

**Table 3:** Summarizing the cost-effectiveness of PCSK9 inhibitors (Evolocumab and Alirocumab) in different countries, including their costs and QALY values.

Country	Drug	Cost	QALY
Germany	Evolocumab	EUR 62,722	0.55
	Alirocumab	EUR 87,002	0.87
United Kingdom	Evolocumab	GBP 45,279	0.53
	Alirocumab	GBP 46,375	0.86
China	Evolocumab	CNY 18,714	1.25
Saudi Arabia	Evolocumab + Statins	USD 60,708	1
	Evolocumab + Ezetimibe	USD 41,757	1
Russian Federation	PCSK9 Inhibitors + Inclisiran	RUB 3.6 million	1

**Table 4:** A comparison of Inclisiran, PCSK9 inhibitors (Evolocumab, Alirocumab), and cholesterol absorption inhibitors (Ezetimibe).

Category	Inclisiran	PCSK9 Inhibitors (Evolocumab, Alirocumab)	Cholesterol Absorption Inhibitor (Ezetimibe)
Mechanism of Action	siRNA silencing PCSK9 gene, reducing PCSK9 production in the liver	Monoclonal antibodies neutralizing circulating PCSK9 proteins	Inhibits cholesterol absorption in the intestine via NPC1L1 receptor
Administration Route	Subcutaneous injection	Subcutaneous injection	Oral tablet
Dosing Frequency	Every 6 months	Biweekly or monthly	Daily
LDL-C Reduction	50–55%	60–70%	15–20%
Onset of Action	Gradual, with sustained effect over months	Rapid effect	Moderate
Compliance	High (due to infrequent dosing)	Moderate (frequent injections required)	High (simple daily dosing)
Side Effects	Injection-site reactions; no significant myopathy or liver toxicity	Injection-site reactions, rare allergic reactions	Mild gastrointestinal discomfort
Ideal Patient Profile	Long-term LDL-C control for ASCVD and familial hypercholesterolemia	High-risk patients needing rapid LDL-C lowering	Moderate-risk patients or statin-intolerant cases
Cost	Typically high (but less frequent injections)	High	Relatively affordable
Relative Advantages	Better compliance due to infrequent dosing	More effective for rapid LDL-C reduction	Non-injectable option
	Lower injection burden compared to PCSK9 inhibitors	Ideal for acute management of post-cardiovascular events	Affordable compared to injectable therapies
			Useful as an add-on therapy with statins
Clinical Indications	ASCVD (for long-term LDL-C management)	High-risk ASCVD patients	Moderate-risk patients
	Familial hypercholesterolemia	Statin intolerance	Statin-intolerant individuals
	Suitable for patients with compliance challenges	Situations requiring fast LDL-C reduction	Add-on for incomplete LDL-C control with statins
